# Detection of Apple Proliferation Disease Using Hyperspectral Imaging and Machine Learning Techniques

**DOI:** 10.3390/s24237774

**Published:** 2024-12-04

**Authors:** Uwe Knauer, Sebastian Warnemünde, Patrick Menz, Bonito Thielert, Lauritz Klein, Katharina Holstein, Miriam Runne, Wolfgang Jarausch

**Affiliations:** 1 Department of Agriculture, Ecotrophology and Landscape Development, Anhalt University of Applied Sciences, 06406 Bernburg, Germany; 2 Cognitive Processes and Systems, Fraunhofer Institute for Factory Operation and Automation IFF, 39106 Magdeburg, Germany; 3 Department of Computer Science and Languages, Anhalt University of Applied Sciences, 06366 Köthen, Germany; 4 RLP AgroScience, 67435 Neustadt an der Weinstrasse, Germany

**Keywords:** hyperspectral, random forest, machine learning, apple, ‘*Candidatus* Phytoplasma mali’, disease monitoring

## Abstract

Apple proliferation is among the most important diseases in European fruit production. Early and reliable detection enables farmers to respond appropriately and to prevent further spreading of the disease. Traditional phenotyping approaches by human observers consider multiple symptoms, but these are difficult to measure automatically in the field. Therefore, the potential of hyperspectral imaging in combination with data analysis by machine learning algorithms was investigated to detect the symptoms solely based on the spectral signature of collected leaf samples. In the growing seasons 2019 and 2020, a total of 1160 leaf samples were collected. Hyperspectral imaging with a dual camera setup in spectral bands from 400 nm to 2500 nm was accompanied with subsequent PCR analysis of the samples to provide reference data for the machine learning approaches. Data processing consists of preprocessing for segmentation of the leaf area, feature extraction, classification and subsequent analysis of relevance of spectral bands. The results show that imaging multiple leaves of a tree enhances detection results, that spectral indices are a robust means to detect the diseased trees, and that the potentials of the full spectral range can be exploited using machine learning approaches. Classification models like rRBF achieved an accuracy of 0.971 in a controlled environment with stratified data for a single variety. Combined models for multiple varieties from field test samples achieved classification accuracies of 0.731. Including spatial distribution of spectral data further improves the results to 0.751. Prediction of qPCR results by regression based on spectral data achieved RMSE of 14.491 phytoplasma per plant cell.

## 1. Introduction

Apple proliferation (AP) is one of the most economically important diseases of apple and is widespread in Europe. It reduces fruit size and quality, which leads to a total yield loss of infected trees [1]. The causal agent, the phloem-limited cell wall-less bacterium ‘*Candidatus* Phytoplasma mali’, is therefore treated as a non-regulated quarantine pest. It is efficiently transmitted by the psyllids *Cacopsylla picta* and *Cacopsylla melanoneura* [2]. As there are no curative treatments against this disease, preventive measures such as uprooting of infected trees and vector control are the only means so far to prevent further spread of the disease. E.g., in some regions of Italy, farmers are made responsible to uproot infected trees by law [3]. For the implementation of these measures, the identification of infected trees on a large scale is of great importance. Although AP induces typical symptoms like witches’ brooms and enlarged stipules [1], these are often difficult to diagnose. Confirmation by molecular means is often required. Large scale monitoring by experts is time-consuming and expensive.

In order to overcome these limitations, the possibility to detect AP by optical measurements was investigated. For this, a secondary symptom of AP, the reddening of infected trees in early autumn, was used. It was shown in a parallel study based on the monitoring of more than 20,000 trees that premature leaf reddening is a highly reliable symptom of AP [4]. It was observed in almost 100% of trees showing specific AP symptoms. In this combination, it has already been regarded as a typical indication for an AP infection [3,5]. Depending on the year of monitoring, ‘*Ca.* P. mali’ was also detected by PCR in 71–97% of trees that showed reddening without any other AP symptoms. Öttl et al. reported a correlation of 86% with AP infection in South Tyrol [6]. AP-related leaf reddening is induced by cold night and warm day temperatures in September [4]. It is the consequence of chlorophyll breakdown induced by phytoplasma infection [7]. Its potential for spectral detection of AP has already been evaluated by Barthel et al., who found differences between infected and healthy ground leaf samples analyzed by near-infrared spectroscopy [8]. However, this is a destructive approach which is still lab-based and labor-intensive. Therefore, Barthel et al. tested a sensor-based method to distinguish AP-infected leaves from healthy leaves in the field with a portable spectroradiometer. By analyzing leaf hyperspectral signatures between 350 and 2500 nm, they were able to identify relevant wavelengths for AP infection [9]. However, this work was conducted only in one infected orchard with one cultivar. It remains uncertain whether the results can be transferred to other orchards with different cultivars and different management strategies.

Biochemical and biophysical modifications induced by pathogens in their host plant have been increasingly used for the development of sensor-based non-invasive detection methods. As phytoplasmas also induce chlorophyll breakdown in many other plant species, spectral variations have already been used for the detection of phytoplasma-infected grapevines. Al-Saddik et al. developed spectral disease indices [10] for the detection of the quarantine pest Flavescence dorée applicable for field detection methods [11]. In Germany, hyperspectral imaging using 400–2500 nm was successfully applied to identify two other distinct grapevine yellows diseases: bois noir and Palatinate grapevine yellows [12].

Spectral measurements are used in many agricultural applications to monitor vegetation health. Several studies showed that hyperspectral sensors are especially valuable tools for disease detection, identification and quantification on different scales from the tissue to the canopy level [13]. Hyperspectral sensors provide a continuous reflectance spectrum of surfaces by measuring reflectance over contiguous, narrow spectral bands in the 400–2500 nm domain [14]. Thanks to their high spectral resolution, these sensors help to determine the spectral bands of interest for a given application, which can be implemented in low-cost multispectral sensors [15]. These multispectral sensors can then be used in satellites, aircrafts or Unmanned Aerial Vehicles (UAVs).

However, the increasing size of hyperspectral data leads to complex data processing and, thus, to higher costs. Therefore, neural networks [16] or machine learning models [17] have been used for data analysis and automated detection. Ali et al. [18] reviewed applications of the most commonly used machine learning algorithms and their advantages and disadvantages. These methods included regression, decision trees, SVMs (support vector machines), artificial neural networks (ANNs), deep learning, and ensemble techniques. Regarding apple, total nitrogen content has been estimated via hyperspectral imaging and machine learning-based regression analysis (partial least-squares regression (PLSR), support vector regression (SVR), and eXtreme gradient boosting regression (XGBoost) [19]. A reduction in the analysis to 5–10 selected wavelengths with the SVR-based prediction model showed a similar or greater performance to that of the full spectrum. So far, only a few studies have addressed the question of disease detection in apple via hyperspectral imaging. Recently, Jiang et al. [20] detected the viral apple mosaic disease by hyperspectral imaging. The disease induces chlorotic spots on the apple leaves, and infected leaves showed a higher reflectance in the range of 500–560 nm.

Hyperspectral measurements are affected by multiple factors, but often only specific wavelength bands are of interest. Therefore, vegetation indices (VIs) have been developed to highlight one factor and reduce the impact of another factor [14]. Vegetation indices are commonly used to measure plant vitality [21], nutrition [22] and biotic and abiotic stresses [23,24]; e.g., the well-established normalized difference vegetation index (NDVI) uses the reflectance of red light and near-infrared light and is correlated to biomass [25], leaf area index [26] and chlorophyll content [27]. Candiago et al. [28] compared multiple VIs like the NDVI and demonstrated that they can be used to detect plant diseases in tomato and grapevine.

To gain insights on the appearance of AP-infected trees and to overcome existing limitations, the spectral signatures of infected and healthy leaves were investigated with hyperspectral imaging under laboratory conditions. The development of a processing pipeline und the utilization of machine learning algorithms aim to derive information about the relevance of certain spectral bands with respect to AP symptoms. This information can then be used to support on-site phenotyping by adapting optical remote sensing technology to the special requirements of AP detection.

## 2. Materials and Methods

### 2.1. Plant Material

Two types of material were used for spectral imaging studies: (1) leaves of ex vitro greenhouse-grown plants infected with a defined strain of ‘*Ca.* P. mali’ and (2) freshly field-collected leaves of AP-symptomatic trees in apple orchards. Ex vitro plants of *M.* x *domestica* cv. Golden delicious were infected with ‘*Ca.* P. mali’ strain PM28, originally transmitted by *Cacopsylla picta* in previous transmission trials [29] and since maintained in micropropagated plants. Healthy plants of cv. Golden delicious served as controls. The plants were part of different experiments to induce AP-related reddening under standardized conditions [4]. Each experiment consisted of 3 infected and 3 healthy plants which were maintained under autumn climatic conditions (12 h cold night at about 5 °C/12 h warm day temperatures at about 20 °C) and a second set of 3 infected and 3 healthy plants which were maintained in parallel under constant warm temperatures (12 h at about 18 °C at night/12 h at about 22 °C at day). At the end of the experiment, 10 leaves per plant were analyzed by hyperspectral and molecular means, whereby this experiment is referred to as AP induction by temperature profile (AP-ITP). Three datasets were analyzed in this study: AP-ITP-1 (induction period 5 September–20 September 2019), AP-ITP-2 (induction period 24 September–9 October 2019) and AP-ITP-3 (induction period 3 September–21 September 2019).

To investigate the variability of leaf-related AP symptoms under natural field conditions, numerous samples from production orchards were collected focusing on older apple orchards with high numbers of AP-infected trees. The orchards were monitored for AP-specific symptoms like witches’ brooms and enlarged stipules as well as for leaf reddening [4]. In 2019, samples from reddening trees were taken in the first half of October. In 2020, samples were taken in the second half of September. Non-symptomatic trees served as controls. All sampled trees were tested by PCR for phytoplasma infection. Table 1 gives an overview of the analyzed orchards and cultivars. In total, 21 different cultivars from 13 different orchards were analyzed, whereby this experiment is referred to as AP field test samples (AP-FTS).

### 2.2. Data Collection

**Figure 1:**
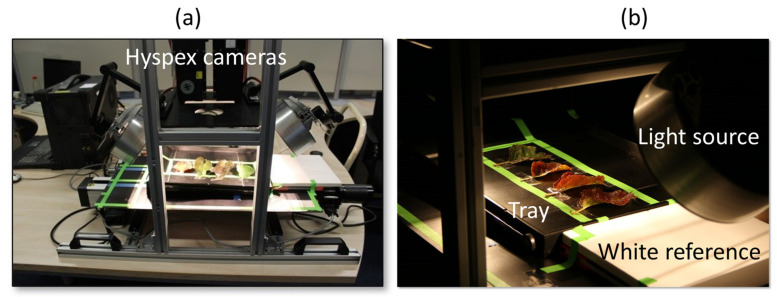
Dual-camera setup at RLP AgroScience. Measurement system with cameras, light sources and moving stage in a rack-mounted setup (**a**) and in operation (**b**).

Following the idea of a non-destructive optical in-field assessment of the disease and its symptoms, data collection consists of customized spectral imaging of the collected plant material. To identify relevant wavelength bands at leaf scale as well as a baseline for subsequent performance evaluation of outdoor measurements at canopy scale, high-quality hyperspectral measurements were carried out under laboratory conditions. The collected spectral data have to undergo several steps of preprocessing. To obtain predictions of disease symptoms, machine learning techniques are applied. The predictions are compared with ground-truth data from molecular analysis of the collected leaves. The imaging approach is shown in Figure 1 and described in the following sections.

### 2.3. Spectral Imaging

Two different hyperspectral dual-camera systems were used as the work was conducted in two different institutes. Both systems consisted of a camera related to the visible to near-infrared region (VNIR) and a camera for the short-wavelength infrared region (SWIR) covering total a spectral range from 400 to 2500 nm.

For the AP-ITP-1 experiment, a fixed dual-camera installation at the Julius Kühn-Institut located at Siebeldingen, Germany, was used. It consists of a Norsk Elektro Optikk (NEO, 1473 Lorenskog, Norway) VNIR-1800 and a NEO SWIR-384 hyperspectral imager. Both cameras are push broom sensors and record single lines of spectral image data at a given frequency. Therefore, the imaging process requires either moving the samples or moving the camera system. Here, a moving table is used (Figure 1).

For the AP-FTS experiment, a mobile dual-camera installation at the RLP AgroScience institute in Neustadt was used. It consists of a NEO VNIR-1600 and a NEO SWIR-320m-e hyperspectral imager. Leaf assays of 10 leaves per plant for the AP-ITP (AP-ITP-2, AP-ITP-3) and 4 leaves of each tree for the AP-FTS experiments have been hyperspectrally recorded for subsequent analysis. Initial preprocessing of the acquired data consists of (1) radiometric correction based on manufacturer calibration data to obtain at-sensor radiance values and (2) normalization with respect to a calibrated white reference plate to obtain at-surface reflectance data. Table 2 lists the specifications of the different cameras. A detailed assessment of the imaging quality and additional key parameters can be found in [30]. Its application is not bound to a laboratory environment [31]. Figure 1 shows the rack-mounted camera setup at RLP AgroScience (VNIR 1600, SWIR 320m-e).

The integration time for each scanline is individually adjusted for the recording of each experiment. Especially, the mobile rack solution used at RLP AgroScience requires this adjustment due to slightly varying positions of the light sources. The recording software Hyspex Ground v3.5 stores the hyperspectral images in ENVI format. Each scan consists of two ENVI images, one for each camera. The software Hyspex Rad v2.0 is then used to obtain at-sensor radiance using a camera specific calibration profile prerecorded at the manufacturer’s laboratory using a light measurement integration sphere.

### 2.4. Image Processing

**Figure 2:**
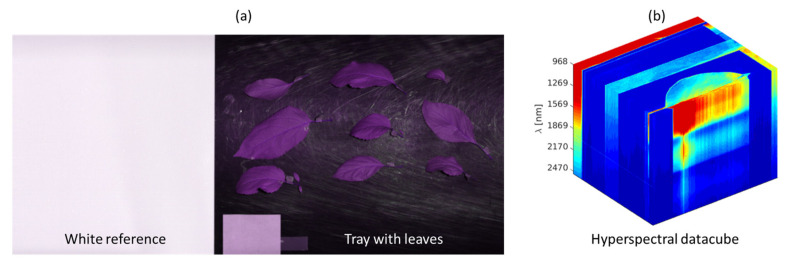
Visualizations of the hyperspectral image data. (**a**) Single-channel intensity image of SWIR 320m-e data with false color information layer (detected leaf area); (**b**) hyperspectral image cube with color-coded radiance data.

Figure 2 illustrates the measured hyperspectral image data. Two different processing chains for data analysis were implemented: first, highly automated processing for fast assessment of the data for analyzing the AP-FTS data, and second, an interactive processing chain to achieve precise segmentation by revision of automated segmentation by human experts to obtain the highest possible accuracy for subsequent steps of the analysis. This processing chain is used to analyze AP-ITP data.

#### 2.4.1. Automated Processing of AP-FTS Data

For testing close to real-world conditions, an image processing pipeline was developed using the C++ programming language and the OpenCV computer vision library. Based on [32], the pipeline extracts spectral data as well as derived spatial–spectral image features for the subsequent machine learning-based data analysis.

First, the position and extents of the white reference panel must be located within the image. Its spectral appearance depends on the camera settings like framerate and integration time as well as on the intensity and spectral characteristics of the used light source. Therefore, an interactive approach ensures that the algorithm can easily be adapted to changing conditions. Within a single channel preview image obtained as the mean intensity of all bands, a scaling value must be selected via a slider control to obtain a visually good separation between the reference plate and the remaining image. The second interactive step consists of selecting a threshold for binarization of the image to obtain good segmentation. Immediate feedback is provided for each step by applying the scaling factor and the threshold online. This interactive procedure can be repeated for the next images until good values are found. Given stable lighting conditions throughout the experiment, well-suited parameters can be found within a few steps. Next, the tool can enter automatic mode to process the complete dataset using the selected parameters.

Next, a set of spectral samples at random locations of an image (N = 1000) on a random subset of the hyperspectral images (N = 15) is drawn. The reflectance of the white reference panel is used to normalize all spectra. Then, dimensionality of the feature space is reduced by applying a principal component analysis (PCA) [33]. A dynamically selected number of principal components is used. Dynamical selection is implemented such that at least 98% of the variance within the spectral dataset must be represented by the subset of principal components. Unsupervised clustering with multiple iterations of the k-means algorithm [34] is used to determine an optimal choice of k. The criterion for the choice of k is minimization of a compactness measure, where the average Euclidian distance between each sample and the center of the corresponding cluster must be determined for different choices of k. For each of the k selected clusters, small grayscale preview images of randomly selected samples are extracted, arranged and presented to the user as a preview of the cluster. The relatively simple task to judge, whether the cluster represents image foreground (e.g., leaves) or background (e.g., white reference plate, metal background), is solved interactively. This constitutes a general pipeline for hyperspectral image analysis where domain knowledge about objects of interests is acquired by asking the user the simple question of whether the set of images represents foreground or background. Based on the acquired reference data, a Random Forest [35] segmentation model is trained and applied to all hyperspectral images afterwards.

After image segmentation into leaf area and image background, individual objects are detected within the leaf area class. A comma separated value (CSV) file is generated, which lists all detected objects within all the images. Unique object identifiers are generated according to an enumeration scheme. Next, the reference data are added to the generated CSV file including either the discrete class information of infections based on PCR or the continuous reference data count of phytoplasma per volume from qPCR results.

Next, a predefined number of spectra are extracted for each object. Again, spectra are stored in CSV format and the given reference data are automatically appended to each spectral sample. The CSV files constitute the datasets used for further processing and data analysis. Derived classification or regression models can then be applied to either CSV stored data or the original hyperspectral images.

#### 2.4.2. Differences and Extensions for the Interactive Processing of AP-ITP Data

For precise interactive preprocessing, a similar approach using a MATLAB-based framework called HawkSpex^®^ Flow is used that has been successfully used in several studies [12,36,37]. Each image of detected leaves is manually validated, and automatically obtained segmentation results are improved using a manual labeling tool. Instead of k-means clustering for the initial image segmentation, Growing Neural Gas [38] is used.

### 2.5. Feature Extraction and Machine Learning

In order to compare different classification algorithms, different datasets are prepared representing different subsets of the conducted experiments and different feature spaces. First, the used features will be described, and second, the choice of ML algorithms is discussed.

#### 2.5.1. Feature Extraction

Prior to evaluation of machine learning, the hyperspectral data are transformed into different feature spaces. In this study, the following feature spaces are investigated:

- Spectral reflectance;
- Normalized spectral reflectance;
- Dimensionality reduced spectral data;
- Spatial–spectral data;
- Selected spectral indices.

Spectral reflectance data consist of the reflectance values for each wavelength band of the hyperspectral image. At-sensor radiance is transformed into reflectance values by division of the radiance value at each pixel by the radiance value measure at the location of the white reference panel. In order to obtain spectral reflectance, the position of the white reference panel in the image must be provided either manually or automatically. Using spectral reflectance data as a feature vector for machine learning enhances the interpretability of the results. Each dimension of the feature space corresponds to a certain wavelength of light, and variable importance measures can be used to determine and understand the physical interaction of the light with the measured materials.

Normalized spectral reflectance is obtained by normalizing each feature vector by its Euclidian norm (L2 norm), which corresponds to the length of the vector. After normalization, all feature vectors have an equal length of 1. Using normalized spectral reflectance is beneficial if the total amount of reflected light differs due to shadows or the bidirectional reflectance distribution function (BRDF) of the material. Again, the wavelength bands constitute the dimensions of the feature space. Therefore, importance measures can also be derived and easily interpreted. However, information about the fraction of reflected light in certain bands is lost when different feature vectors are compared and interpretation becomes more difficult.

Dimensionality-reduced spectral data are based on PCA. It projects the dataset into a low-dimensional feature space, which preserves most of the variability of the original dataset. Each dimension of the novel feature space is a linear combination of all original dimensions. It is an orthogonal transform which ensures linear independence of the principal components (base vectors of the new feature space). As the novel features are a linear combination of the original features, it is more difficult to interpret the feature values. The PCA outcome depends on the used dataset, and the transformation matrices might change dramatically if new samples are integrated into the dataset.

Spatial–spectral data integrate the spectral information and data from neighboring pixels. Different approaches exist. Linear discriminant analysis (LDA) was used to preserve class-specific spectral information. The high-dimensional feature space of spectral data is transformed into a low-dimensional feature space, where each dimension is an optimized feature with respect to separation of one of the classes. Each new dimension is a linear combination of all original dimensions, and therefore, important information of the spectral data is preserved in the low-dimensional feature space. Spatial information is integrated by calculating different statistical measures for different image blocks centered around the image position of the feature vector using the three different statistical measures of mean, standard deviation and homogeneity as well as the first three components of the LDA and 25 different block sizes from 4 × 4 to 100 × 100 pixels. The resulting feature space has 225 dimensions. Therefore, better would be This results in a 225 dimensional feature space. Using spatial–spectral features intends to integrate information about the spatial distribution and variation in object reflectance into the pixelwise decisions of a classifier. Therefore, it adds additional robustness to the decision-making. However, as the information from the neighborhood becomes more important, small regions of interest might be more difficult to detect by a classifier.

Spectral indices aim to reduce the dimensionality of spectral data and to allow for high interpretability of the feature values. Typically, a spectral index comprises only a few spectral bands to calculate a problem-specific measure. Most spectral indices originate from the field of remote sensing and address properties of vegetation, soil, water bodies and other surfaces. The spectral indices NDVI, PRI, CCI and GLI were selected, based on the ability to detect various plant stresses (NDVI), levels of vegetation health (PRI) and chlorophyll contents (CCI, GLI). Definitions and references of spectral indices are provided in Table 3. Using spectral indices provides a highly interpretable feature space but limits the information to only a few bands. This yields the chance to miss important features when applied to new classification problems.

#### 2.5.2. Machine Learning

For the evaluation of different machine learning approaches, MathWorks Matlab (version 2024a) is used. Evaluation uses two different approaches:

- Experiments using own implementations of ML algorithms;
- Experiments using the suite of provided ML algorithms.

The first approach uses a self-developed framework that has been described in Section 2.4.2 for interactive preprocessing. The second approach uses the integrated Matlab apps Classification Learner and Regression Learner of the Statistics and Machine Learning Toolbox. Both kinds of experiments aim to achieve the following objectives:

- Identify the most relevant wavelength bands for detection of apple proliferation;
- Identify the best feature space–classifier combinations to solve the detection problem;
- Better understand the options and limitations of automated diagnosis of the disease;
- Propose an approach for implementation of disease detection.

From the set of available methods, the following ML algorithms have been selected for investigation:

- Decision trees with different levels of pruning;
- Ensemble methods based upon decision trees;
- Support vector machines with different kernels;
- Neural networks with different topologies.

To compare results, n-fold cross-validation (n = 10) is used in an integrated training and testing approach. Stratified datasets are used and mean classification accuracy, confusion matrices and ROC curves are used as means to evaluate classification performance. Root mean square error (RMSE) is used as a measure to compare different regression methods for prediction of disease levels. Based on the results of the experiments, the Random Forest classifier, as a fast-to-train and general-purpose ensemble classification method, has been implemented into the automated processing pipeline described in Section 2.4.1 for analyzing the AP-FTS experiment.

### 2.6. PCR Analysis

For the AP-ITP experiment, each leaf was tested individually for phytoplasma infection. For the AP-FTS experiment, the branch from which the leaves were sampled was tested. Total nucleic acids were extracted either from leaf petioles (AP-ITP) or from phloem preparations of branches (AP-FTS) according to [29] using a CTAB-based protocol. For phytoplasma detection, European fruit tree phytoplasma-specific primers fO1/rO1 were used as published in [44]. Aliquots of each PCR product were analyzed by agarose gel electrophoresis.

The phytoplasma titer was analyzed in the AP-ITP experiment in the individual leaves by quantitative PCR as described in [4]. Specific primers AP3/AP4 were used for absolute quantification with the standard curve method according to Jarausch et al. [45]. These data were normalized with the absolute quantification of an apple single-copy gene according to Liebenberg [46]. Phytoplasma concentration in each leaf was thus expressed as phytoplasma copy per plant cell.

## 3. Results

In this section, the results of different experiments with the AP-ITP and AP-FTS datasets are provided. First, detection of AP infections with regression methods as automation of the lab-based PCR approach is presented for the AP-ITP experiment. Second, results of binary classification (infected vs. healthy) for the AP-ITP experiment are presented. This includes assessment of the relevance of spectral bands for solving the classification problem. Third, existing spectral indices are investigated as a means to provide reliable disease detection. Fourth, the performance of different ML algorithms for disease detection using spectral data from the complex AP-FTS dataset is presented. Finally, possible improvements by using spatial–spectral features instead of spectral data are reported.

### 3.1. Regression of qPCR Values to Estimate Infection Levels (AP-ITP-1)

**Figure 3:**
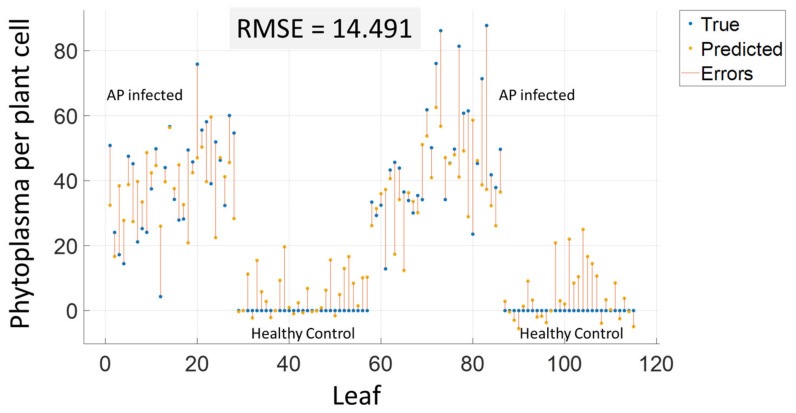
Prediction of qPCR values based on regression. The mean spectrum of individual leaves from the VNIR camera served as predictors, while the corresponding qPCR results for the leaves served as the target variable.

Infection with AP was validated with qPCR for the experiment AP-ITP-1. That dataset was used to evaluate the potential of different regression algorithms to predict the qPCR value. Figure 3 shows the validation plot of the best-performing method.

Table 4 lists the results of different regression algorithms. The best RMSE, as presented in Figure 3, was achieved by limiting the number of input features to the 20 most important wavelength bands using the RReliefF algorithm [47], further reducing dimensionality by PCA to 10 features, and using Gaussian Process Regression. For all other regression algorithms, the table reports the results of similar feature reductions as well as the individually best-performing approach of each alternative approach. For Gaussian Process Regression, results within other feature spaces and for different feature selection algorithms are reported to provide a good summary of the prediction quality.

The response of the regression method was obtained by 10-fold cross-validation. The results indicate that prediction of medium concentrations is better than prediction of high concentrations and detection of healthy leaves. Based on the obtained RMSE value, a detection threshold can be defined to minimize false detections. In addition, classification of the symptoms can be combined with the regression method in a hierarchical approach to improve the results.

### 3.2. Feature Relevance and Classification Performance Using Spectral Data in Controlled Experiments (AP-ITP-2, AP-ITP-3)

**Figure 4:**
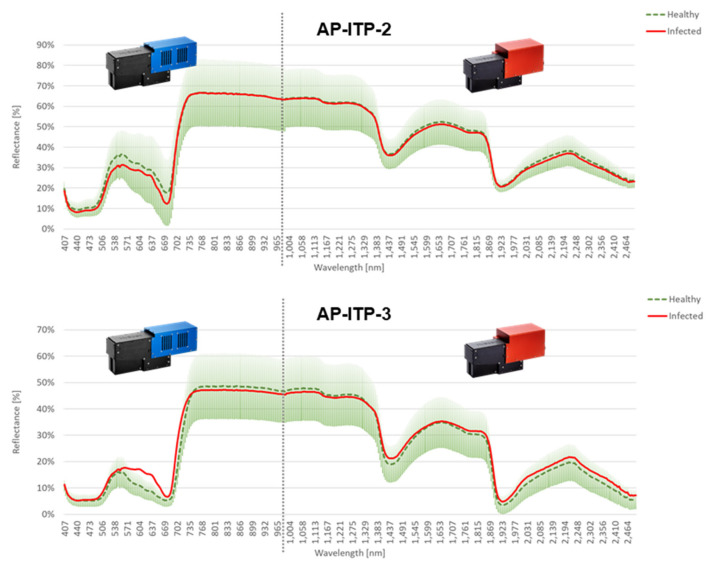
Mean spectra of healthy and infected leaves with per-band standard deviation of reflectance plotted for experiments AP-ITP-2 and AP-ITP-3.

The comparison of the measured spectral data for AP-ITP-2 is shown in Figure 4. For the healthy leaves, the standard deviation of reflectance is plotted in addition to the average spectrum. The mean spectrum of infected leaves clearly shows a difference between the two classes in the spectral bands between the so-called green peak (~550 nm) and the beginning of the so-called red-edge region (~700 nm).

In contrast to the visible and near-infrared bands, no clear difference is visible in the shortwave infrared region, given the high standard deviation within the pixels of the healthy leaves. Also, in the experiment AP-ITP-3, the differences in the mean spectra concentrate in the visible region between the green peak and the start of the red edge (Figure 4). However, differences in the reflection of green light are observable in lower bands compared to AP-ITP-2. Also, the differences in the shortwave infrared region and the water absorption bands are visually less significant.

Diagrams in Figure 4 provide a visual cue as to which bands are of special interest for an automated diagnosis of apple proliferation disease by hyperspectral remote sensing. The presence of a high standard deviation for the measured reflectance per pixel within these bands highlights the need for more sophisticated classification approaches. Table 5 contains the confusion matrix for the classification of VNIR and SWIR data using an Auto-ML-approach with the 10-fold cross-validation of the best-performing ML algorithm. The used dataset is balanced with the same number of spectra of infected and healthy leaves. Spectra are randomly selected from the full dataset.

**Figure 5:**
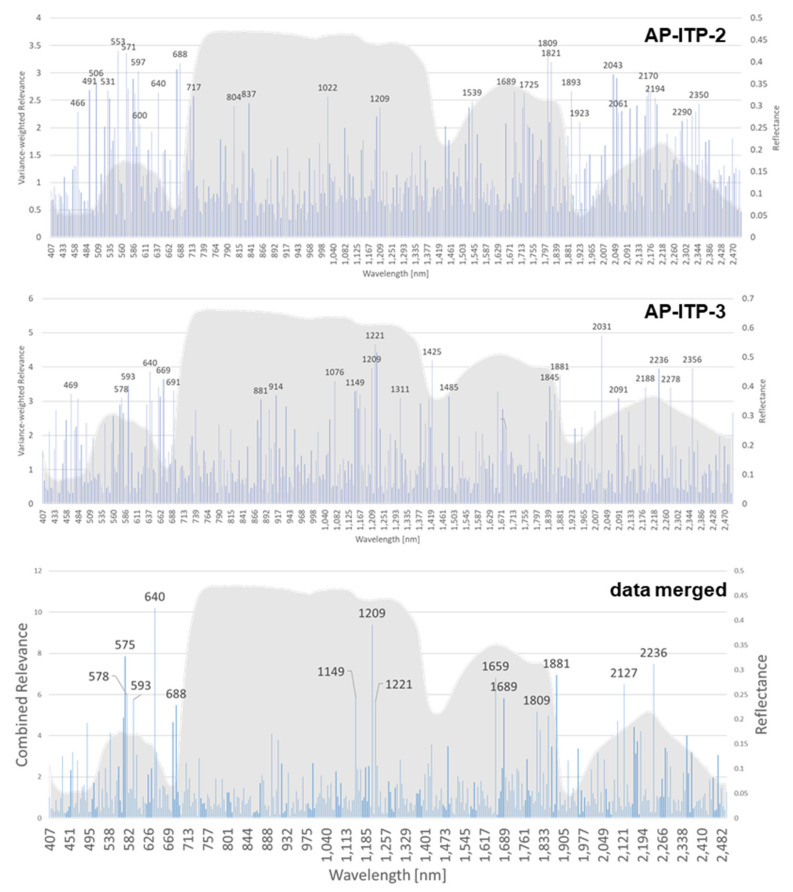
Variance-weighted relevance of individual bands for the rRBF classifier of the AP-ITP-2 and AP-ITP-3 datasets. In addition, the combined relevance of individual bands for the rRBF classifier of both datasets is shown.

The rRBF classifier yields a weight vector for the input features as an additional result of model training. This can be interpreted as the relevance of input features and as a starting point for reducing dimensionality and model complexity. As 10-fold cross-validation results in 10 different models, the variance of the weights is also obtained. It is used to weight the mean relevance of each band. Bands with high variance of relevance in 10-fold cross-validation receive a lower weight and vice versa. Relevance values for AP-ITP-2 and AP-ITP-3 are shown in Figure 5. The figure shows the variance-weighted relevance of individual bands in the decision process of the rRBF classifier.

For both AP-ITP-2 and AP-ITP-3, the results indicate the importance of the complete spectral information to achieve the classification accuracy of 0.971 in cross-validation. Also, the high number of relevant bands indicates the complexity of the detection problem. These insights, that the machine learning approach provides the relevance measure, help us to judge the importance of the visible differences in the mean spectra in Figure 4. By multiplying the band-specific relevance measures of AP-ITP-2 and AP-ITP-3, bands of general interest and relevance for detection of an AP infection in both experiments can be emphasized. Figure 5 also shows these results. Five bands in the visible part of the electromagnetic spectrum (575 nm, 578 nm, 593 nm, 640 nm, 688 nm) and nine bands in the NIR and SWIR part (1149 nm, 1209 nm, 1659 nm, 1689 nm, 1809 nm, 1881 nm, 2127 nm, 2236 nm) are of special interest. Here, a threshold of 60 percent of the maximum relevance measure was used to discriminate between relevant and less relevant bands.

### 3.3. Spectral Indices as a Means for Dimensionality Reduction and Data Visualization

**Figure 6:**
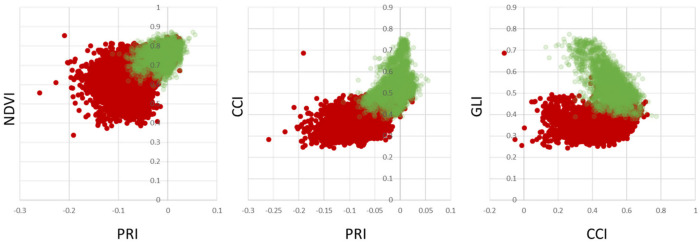
Scatterplots showing selected spectral indices of pixels from healthy (green) and infected (red) leaves.

Spectral indices are a means of dimensionality reduction in spectral data analysis by preserving a high level of information with respect to common traits in monitoring the vegetation and the environment. Figure 6 shows scatterplots of healthy vs. infected leaves per pixel. The overlap of the point clouds within the scatterplots indicate that healthy plant material within infected leaf tissue limits the ability to discriminate between healthy and infected leaves. However, the distribution of pixels belonging to healthy trees clearly indicates a unique distribution within the three feature spaces shown. As PRI, NDVI, CCI and GLI are commonly used vegetation indices to detect plant stresses, chlorophyll concentration and senescence, the results provide support for their potential application to detect apple proliferation disease.

**Figure 7:**
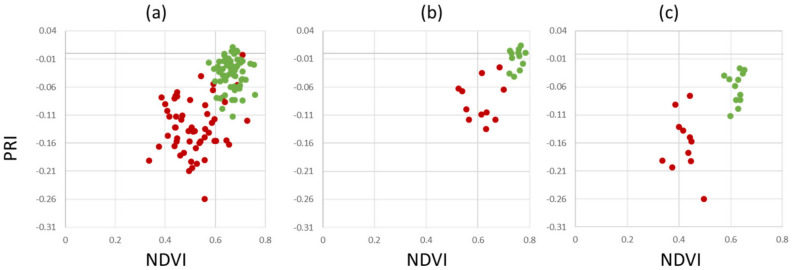
Healthy (green) vs. infected (red) scatterplots of aggregated spectral indices: (**a**) minimum value per leaf, (**b**) average value per tree and (**c**) minimum value per tree (AP-ITP-2).

To overcome the limitations of separability, different measures that can easily be implemented into automated disease detection algorithms have been investigated. Figure 7 shows the results for the dataset AP-ITP-2. Using the minimum NDVI value per tree (condition c, where an assay of five leaves per tree has been used) provides a reliable separation between healthy and infected trees. Using the average NDVI value in combination with average PRI (condition b) already provides clear distinction between classes and is assumed to be more robust to outliers. However, if diagnosis is based on single leaves, false classifications still persist (condition a).

**Figure 8:**
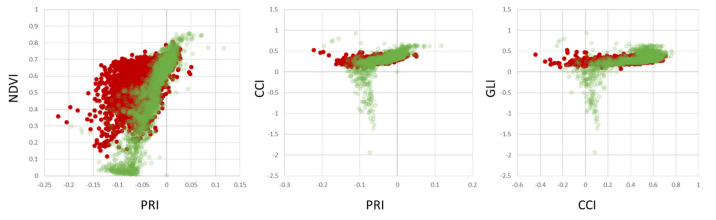
Healthy (green) vs. infected (red) scatterplots of selected spectral indices per pixel for experiment AP-ITP-3.

Validation of the findings from AP-ITP-2 with a different experiment (AP-ITP-3) yields different results with respect to the value distributions and separability of healthy and infected leaf pixels. Figure 8 shows PRI, NDVI, CCI and GLI scatterplots.

**Figure 9:**
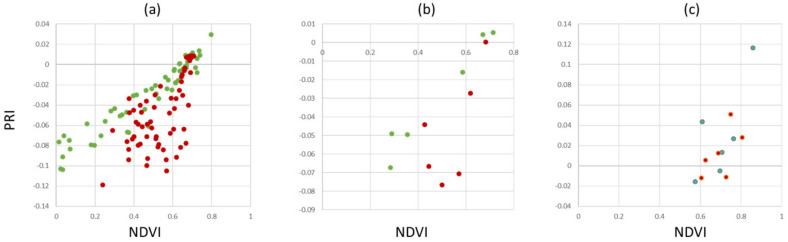
Healthy (green) vs. infected (red) scatterplots of aggregated spectral indices: (**a**) minimum value per leaf, (**b**) average value per tree and (**c**) minimum value per tree (AP-ITP-3).

The aggregation of the pixelwise measurements using mean values per leaf as well as per tree improves the performance (Figure 9). However, using the minimum value for the spectral indices NDVI and PRI does not improve the results.

### 3.4. Classifier Performance and Model Selection Using Spectral Data and Samples from Orchards (AP-FTS)

The next step is the integration of classification models into a highly automated AP detection pipeline. Therefore, different machine learning approaches are investigated for their performance with automated feature extraction (without manual revision of the extracted data). The spectral data, which constitute the training set, are obtained by random sampling from the hyperspectral images. First, they are normalized by L2-norm and then used as input to a suite of classification algorithms for direct comparison of the classifier performance. Stratified sampling was used within each hyperspectral image. Because trees with apple proliferation and healthy trees are not equally represented within the images, stratification is applied to provide an equal number of spectra for the two classes. Table 6 summarizes the classification performance of the selected ML algorithms. For the 2019 measurements, the Hyspex SWIR camera in combination with the SVM classifier provided the best results. For the 2020 measurements, again, the SWIR camera yields better results, but in combination with the Random Forest classifier. The results of the Hyspex VNIR camera are slightly less reliable than SWIR data for both years. Either way, ensemble methods or SVM classifiers yield the best results.

**Figure 10:**
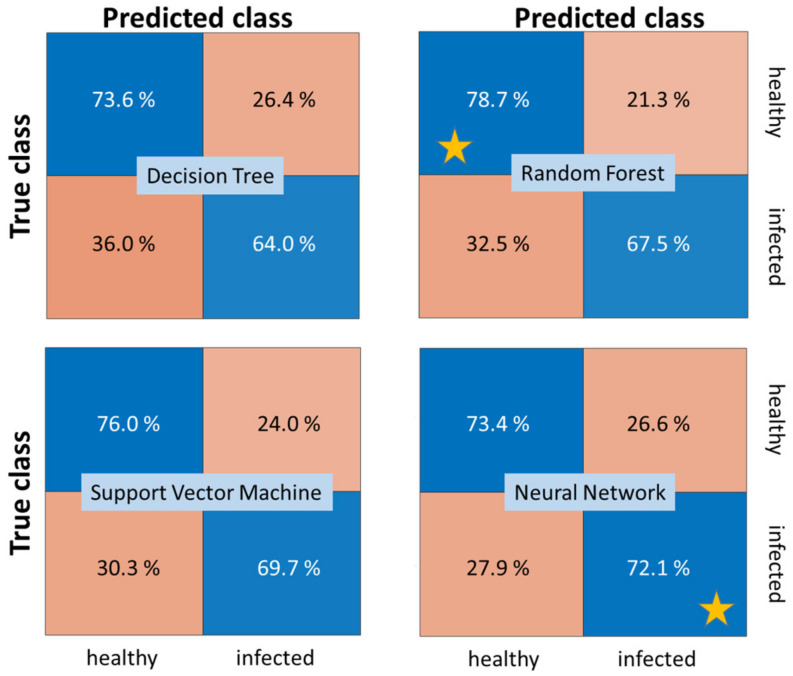
Comparison of confusion matrices for 2020 SWIR dataset AP-FTS. The stars denote the best obtained TPR for the two classes. Operating points correspond to equal misclassification cost. Models correspond to best-performing models from Table 6 (last column) for each method category.

Figure 10 shows confusion matrices for the best-performing variants of each group of methods in Table 6 to gain more insights into the classification performance and differences among the classification approaches. While the Random Forest classifier yields the best overall performance, it also minimizes the number of false detections of an infection compared to all other methods. However, the MLP neural network classifier is able to detect more of the true infections and nearly matches the overall performance of the Random Forest.

**Figure 11:**
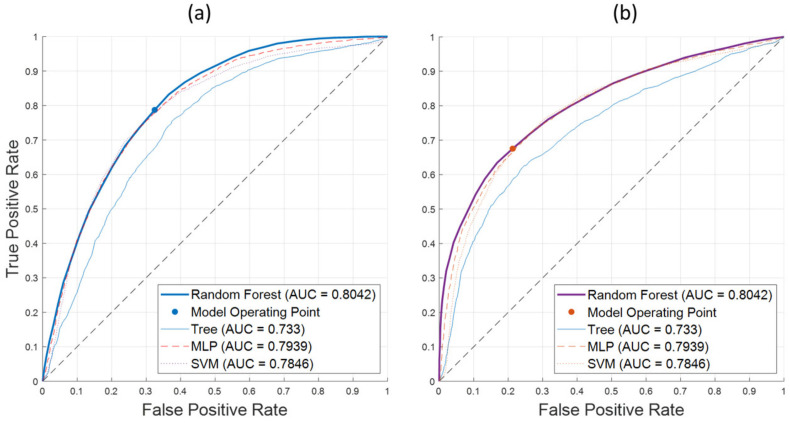
ROC curves for best-performing ML algorithms on the 2020 SWIR dataset AP-FTS. (**a**) ROC curve for detection of healthy leaf tissue; (**b**) ROC curve for detection of leaf tissue of infected trees. Operating points correspond to equal misclassification cost. Models correspond to best-performing models from Table 6 (last column) for each method category. Random Forest classifier outperforms other models in the ROC space as indicated by maximum AUC measure.

Figure 11 shows ROC curves for (a) the detection of spectra from healthy leaf tissue and (b) the detection of spectra from leaf tissue of AP-infected trees. Again, the Random Forest classifier shows its superior performance over the full range of possible operating points. This corresponds to the AUC measure of 0.8042. The operating points correspond to equal classification costs and the accuracy values in Table 6.

Given that the Random Forest classifier yields the best performance in the SWIR 2020 dataset and overall good ranking among the other datasets (second rank among all datasets), it was chosen for integration into the automated detection pipeline.

**Figure 12:**
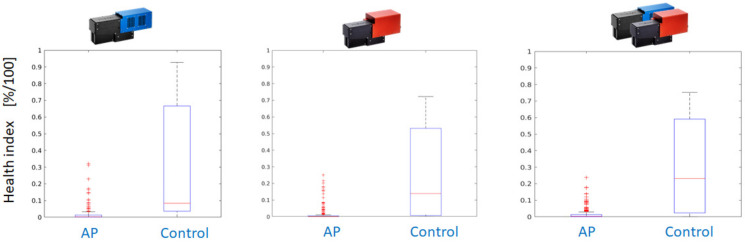
Boxplots with a comparison of disease detection quality (from left to right) for VNIR, SWIR, and combined decision based on the fraction of affected surface used as a health index.

As a consequence, the application of the Random Forest classifier yields pixelwise classification results for all available AP-FTS images from the years 2019 and 2020. As the degree of reddening and the affected leaf surface differ between different varieties and the date of sample collection, a health index is calculated from the pixelwise decisions. The health index is the fraction of healthy leaf surface based on pixelwise decision of the Random Forest classifier. Figure 12 compares the performance using measurements with Hyspex VNIR only, Hyspex SWIR only, and both cameras.

**Figure 13:**
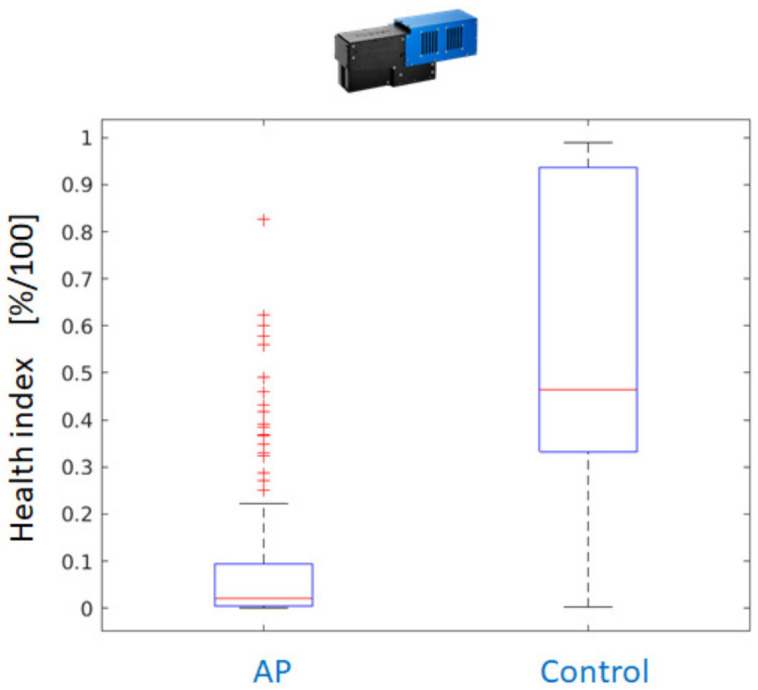
Separation between healthy and AP-infected trees is improved by digital image processing (DILATION with a 5 × 5 rectangular kernel to emphasize healthy regions).

Postprocessing is applied to further improve the results. It consists of a morphological operation (dilation with 5 × 5 rectangular kernel) on the label image with classification results. Even for a single-camera system operating in the VNIR region, the dilated classification maps outperform the dual-camera setup. Figure 13 shows the better separation of health index values between AP-infected leaves and healthy controls for the VNIR camera. If a threshold of health index 0.25 is applied, only a few leaves of infected trees are falsely classified as healthy and the majority of leaves of healthy trees can be correctly classified.

**Figure 14:**
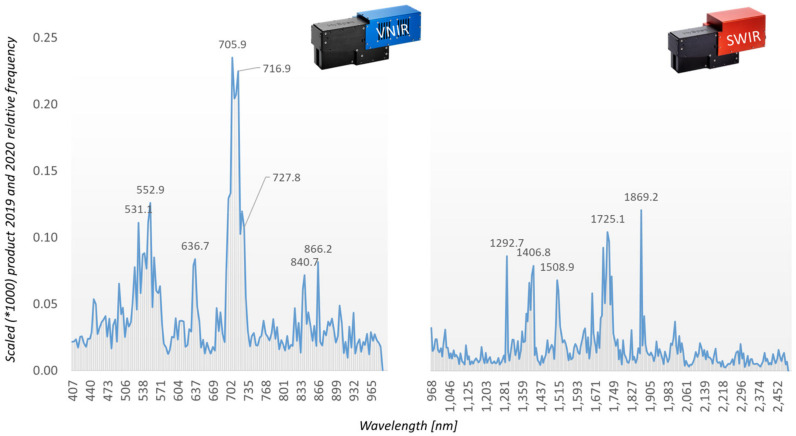
Relevance measure of individual bands (frequency of use in decisions for the whole dataset) obtained for AP-FTS.

The field test samples (AP-FTS) in combination with the Random Forest classifier yield additional insights into variable importance. Figure 14 shows the frequency of assessing a certain band in the decisions of the Random Forest.

**Figure 15:**
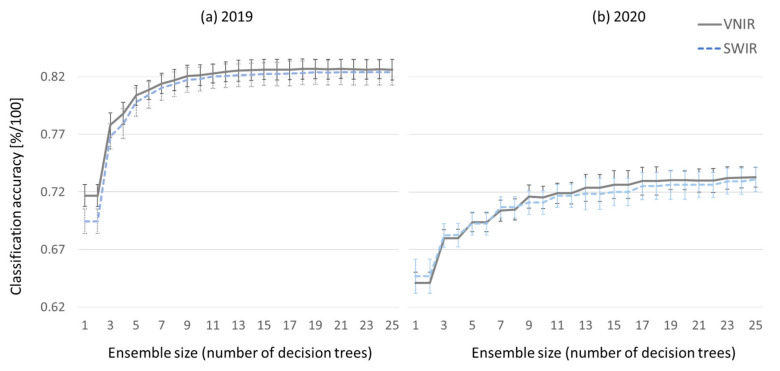
Pixelwise classification accuracies (infected vs. healthy leaf tissue) for different numbers of decision trees in Random Forest classifiers show better classification performance for AP-FTS data from VNIR hyperspectral camera and all ensemble sizes in years (**a**) 2019 and (**b**) 2020.

Figure 15 shows the classification accuracies and their standard deviations for different sizes of the Random Forest ensemble classifier. With increasing ensemble size, the classification accuracies improve.

**Figure 16:**
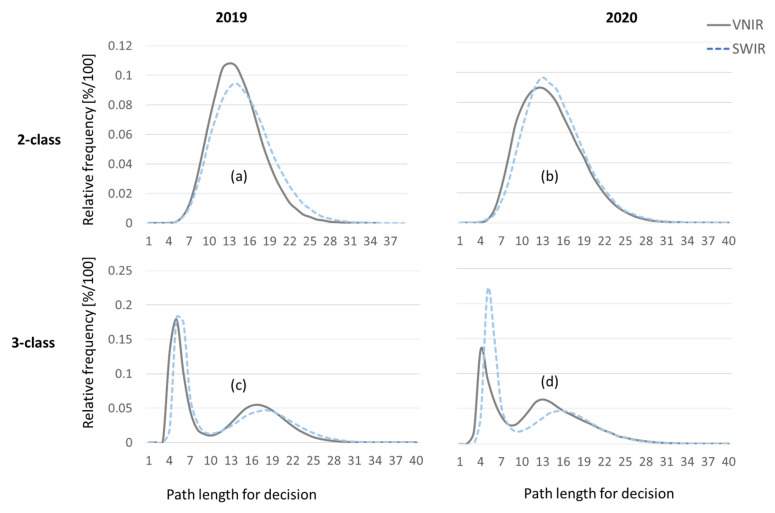
Complexity of decision indicated by path lengths (wavelength bands evaluated during a single decision) of unpruned decision trees. Using VNIR data, decisions are found with less comparisons in both years, 2019 and 2020, compared to SWIR data. Complexity of classification of infected vs. healthy pixels for (**a**) 2019 and (**b**) 2020. Additional detection of background as 3rd class (**c**,**d**) requires less operations than classification of leaf tissue (bimodal distribution).

To investigate which camera and which range of the electromagnetic spectrum yield better results, VNIR and SWIR camera data are processed independently. Figure 16 compares the complexity of the pixelwise decisions based on the number of bands per tree that are evaluated for each decision. More difficult decisions correspond to longer path lengths in the decision trees that constitute the Random Forest. Also, longer decision paths from root node to the leave nodes of the decision trees correspond to higher computation times, both in classifier training and classifier recall. The left column of Figure 16 shows results for the 2019 dataset and the right column for the 2020 dataset, respectively. The upper row shows pathlength distribution for the two-class problem to discriminate between infected and healthy leaf spectra. As the Random Forest classifier is able to efficiently tackle multi-class problems, the histograms in the lower row represent the three-class problem to discriminate between the two leaf classes and the image background. Both diagrams for the three-class problem exhibit a bimodal distribution. The first peak corresponds to detection of the background pixels. Because separation of leaf tissue and metal background is the easier problem, the number of wavelengths to be evaluated is low compared to number of bands used for discrimination between infected and healthy leaf area.

### 3.5. Evaluation of Spatial–Spectral Classification for Leaf Samples from Orchards (AP-FTS)

A problem-specific dimensionality reduction with LDA and statistical measures for image blocks of different sizes provide an alternate feature space for image segmentation. Table 7 lists the classification accuracies based on this feature space. Classification of the 2019 dataset is improved by more than 6 percent for both VNIR and SWIR camera data. For the 2020 dataset, no major improvements are observed. Using spatial–spectral features, classification accuracy for both years reaches a similar quality (2019 = 0.751, 2020 = 0.734, see Table 7) compared to pure spectral classification (2019 = 0.685, 2020 = 0.731, see Table 6).

## 4. Discussion

In this article, results of ML-based detection of apple proliferation disease were reported. Hyperspectral imaging was used as a means to record data under controlled conditions of illumination and positioning of the leaves. This setup might be used as an additional lab-based test for diagnosis of the disease, e.g., in a mobile laboratory. It can potentially outperform PCR tests in terms of sample preparation, required time for testing and sample throughput. Therefore, this study aims to report on the detectability of visible (e.g., leaf reddening) and invisible symptoms of apple proliferation. Moreover, the results are expected to support the implementation of proximal diagnosis in the field by providing information about relevant wavelength bands and an upper bound on expected detection quality given optimal imaging conditions in the laboratory environment.

Analysis of samples from the AP-ITP experiments, where apple trees have been artificially infected and expression of symptoms has been studied under different conditions in a climate chamber, showed the highest level of detectability using classification algorithms (AP-ITP, a mean accuracy of 0.971 in 10-fold cross-validation of leaf spectra per pixel with the rRBF classifier). However, these experiments were limited to a single apple variety, and both inoculation and measurements took place in a controlled environment. A limited generalization of the results to real-world samples is supported by the findings on spectral indices and the separability of infected and healthy tissue. Based on a comparison, the AP-ITP-2 and AP-ITP-3 samples exhibit very different distributions of index values for NDVI, CCI, GLI and PRI between the experiments. Within each experiment, a good separation of infected and healthy samples is observed for using mean indices per tree and per leaf instead of indices per pixel. This reflects the fact that leaf symptoms of apple proliferation are not equally spread over the leaf and are differently distributed on leaves of a single infected tree. Therefore, results of pixelwise classification might always be biased towards false detections of the disease, as healthy tissue is present on leaves of infected trees and therefore is potentially included in any training sample. To address this intrinsic problem, several measures have been investigated: using features of leaves and trees instead of pixelwise features, using spatial–spectral features, and using morphological operations on label maps after pixelwise classification. All mentioned techniques improve results when integrated in the decision process and can be used in combination.

In spectral imaging of vegetation, coupling exists between the pattern of reflected light at certain wavelengths and physiological processes and reactions to biotic and abiotic stresses [14]. Therefore, the relevance of input features for solving a classification or regression problem allows us to analyze underlying processes and differences between classes. For the AP-ITP experiment, the feature relevance after training an rRBF classifier was extracted. Because 10-fold cross-validation results in 10 different models, the standard deviation of the relevance value was incorporated as an inverse weighting factor. Because neighboring bands of the reflection spectrum of vegetation are highly correlated, random effects like the elements within each fold of the training dataset and random choices in classifier training like initialization parameters lead to slightly different positions of most relevant bands. Therefore, the results of AP-ITP-2 and AP-ITP-3 were combined into a generalized relevance profile in Figure 5 by multiplying the obtained and weighted relevance profiles of the two datasets. This approach better exploits the spectral bands of general interest for AP detection. So far, the classification performance achieved by limiting the input features to a subset of the most relevant bands has not been investigated. Interestingly, the most relevant bands of the VNIR camera concentrate in the region of visible light. Thus, operating an RGB camera with high spatial resolution for AP detection seems feasible. However, the decision-making process itself uses all available bands, and the relevance profiles for the individual datasets show that the NIR and SWIR bands are also important.

For the AP-FTS experiment, the variable importance was estimated by analyzing the decision process within the individual decision trees of the ensemble classifier. The histogram of accessing a certain wavelength band is recorded and used as an importance measure. The relevance profiles of the different years 2019 and 2020 have been combined in a similar way as described for the AP-ITP experiment (Figure 14). The locations of the most relevant bands obtained are strongly correlated with well-known properties of the reflectance spectra of vegetation, namely the green peak at ~550 nm, a red band at ~637 nm, a number of bands in the red-edge region centered at ~706 nm, and NIR bands at ~840 nm and ~866 nm. Similar but slightly different results were obtained by [9] while analyzing leaf samples in the field. In October, relevant wavelength ranges for AP detection were 499–521 nm and 668–700 nm. The data of [9] were obtained with a spectroradiometer analysis of only one single variety, which explains the slightly better accuracy of 0.870. By contrast, the AP-FTS dataset consists of samples from many different orchards and apple varieties. Therefore, the obtained results are promising for application of proximal sensing of all AP-infected varieties with multispectral camera systems, either ground-based or airborne. The relevance peak at ~637 nm might be correlated to reports of a special red color which a trained human expert can detect on infected trees. It must be noted that the good classification results depend on more than a single feature, but the design and performance of specialized camera systems for AP detection can benefit from the requirements of the detection algorithms.

Another concern is year gap influences that were observable especially in the relevance profiles. So far, this year gap information helped us to identify the intersection of relevant wavelength bands between years. Potentially, this information can be used to apply multispectral cameras adapted to the most relevant wavelength bands or to train a classifier adapted to selected bands only. The classification accuracies for 2019 and 2020 can be interpreted as the upper bound of the achievable classification performance given the approaches presented and validated here. A combined model based on data from both years would yield either similar or less accurate results (especially due to different varieties collected in 2019 and 2020, respectively; see Table 1). However, the model chosen for operational use should always include the available data completely.

A subset of available classification algorithms was selected to solve the AP detection problem. SVM with different kernels, ensemble classifiers, and the MLP neural network classifier still represent state-of-the-art classifiers for the task of spectral data classification. Single decision trees have been included as control, because the used ensemble classifiers consist of a number of decision trees. So far, deep learning approaches like CNN classifiers have not been investigated for the given problem. If the available training dataset is expanded, this could be a promising direction for future research. Such research should focus on providing pretrained models for different plant diseases which can then be adapted to novel use cases. However, interpretation of the decision process and relevant features becomes more complicated as model complexity is drastically increased.

In addition to the detection of an AP infection based on hyperspectral imaging of the leaves, the regression of qPCR results based on hyperspectral data was investigated. While, the achieved RMSE of ~14.5 phytoplasmas per plant cell is promising, spectral data of healthy leaves receive relatively high response values of up to ~25. Therefore, the optical assessment is too sensitive compared to qPCR. If these results are combined with a detection threshold and aggregated from leaf to tree level by averaging the results of qPCR as well as the optical measurements, a further improvement can be expected. Barthel et al. [9] analyzed the phytoplasma colonization in apple trees in the field from April to November and measured the phytoplasma concentration in leaf samples. They identified specific bands in their hyperspectral imaging (e.g., 500 nm) for which the classification results improved with the phytoplasma concentration in the leaves in October and November when leaf reddening is most expressed. The above results indicate that the efficiency of AP detection by spectral methods is depended on the phytoplasma concentration.

A remaining uncertainty is the question of how closely the local concentration of phytoplasma and infection symptoms like reddening of a leaf are correlated. A previous study has shown that the intensity of leaf reddening is not correlated to the phytoplasma titer in the leaf petiole and concluded that leaf reddening is a long-distance effect of the phytoplasma infection of the tree [4]. As phytoplasmas are not homogenously distributed in AP-infected trees, molecular detection may be hampered by the small sample size used for diagnosis. In this regard, optical assessment of reddened leaves can be more sensitive than molecular diagnosis [4].

Phytoplasma infection weakens the tree and induces a multitude of physiological changes. Therefore, specific and unspecific hyperspectral effects are difficult to distinguish and common indices for growth parameters like NDVI already achieve relatively good results. Different VIs have already been applied for the diagnosis of plant diseases [28]. Phytoplasma infection induces premature chlorophyll breakdown [7] which leads to specific leaf reddening due to the presence of the anthocyanin cyanidin-3-glucoside [48]. This symptom is totally different from leaf senescence in fall and is highly correlated to the presence of the phytoplasma [4]. Therefore, the results pave the way for a large-scale hyperspectral diagnosis of AP in the field, either in a mobile laboratory or with a portable spectroradiometer, as performed by [9]. However, outdoor measurements face certain challenges such as varying illumination and observation angle as well as individual leaf segmentation. In proximal sensing strategies with a spectroradiometer or a vehicle-based setup [49], internal light sources are used to guarantee homogenous illumination. Novel segmentation approaches using convolutional neural networks can solve the segmentation problem. The results of the present study show that disease detection improves with averaging by considering decisions of multiple leaves per tree. So, the challenges can possibly be addressed by segmentation of leaf area (not individual leaves) against background (stem, soil).

The hyperspectral research of AP symptoms is also a prerequisite for establishing remote sensing strategies for AP. The unique camera perspective limits its applicability and the direct transfer of the models from the laboratory. However, identified wavelength bands with high relevance in decision-making support the choice of spectral bands in multispectral imaging. It is necessary to combine UAV-collected data (NADIR view) with reference data from experts and lab analysis to create sophisticated classification models. Remote sensing of AP with multi- or hyperspectral data obtained by UAVs or satellites has already been attempted [50].

## 5. Conclusions

In this paper, the results of a 2-year study of leaf symptoms of apple proliferation (AP) are reported. They provide the foundation of future research and development towards in-field assessment of AP by optical sensors. The most relevant wavelength bands were identified based on machine learning, which can lead to an optimized design of measurement instruments for AP detection. A suitable processing pipeline for hyperspectral images has been proposed, and different parameters to improve classification results have been investigated. The use of spatial–spectral features further improves detection quality. Detection quality also benefits from classification of leaves instead of pixels as well as further aggregation of the decisions to decisions per tree.

## Figures and Tables

**Table 1:** Origin, cultivar and health status of apple leaf samples analyzed in AP-FTS. Samples are grouped by the two regions Vorderpfalz and Südpfalz (bold/italics) where they have been collected. Total numbers of samples are provided in the last line (bold).

Region		2019		2020	
Orchard Code	Cultivar	N° Symptomatic Leaves	N° Healthy Leaves	N° Symptomatic Leaves	N° Healthy Leaves
***Vorderpfalz***					
Nied1	Golden Delicious	12	4	32	8
NW5	Rubinette	40	4	8	16
NW13	Ambasie	8	4		
NW13	Axam	16	0		
NW14	Delbarestivale	24	0	16	36
NW14	Falstaff	20	0		
NW14	Jonagold	16	0	4	4
NW14	Pink Lady	20	0		
NW14	Pinova	20	4	16	16
NW14	Rubinola	20	4	4	24
NW14	Topaz	52	4	44	8
NW16	unknown	72	24		
NW17	Berlepsch	24	0		
NW17	Boskoop	36	4		
NW17	Fuji Yataka	12	0		
NW17	Gala	16	0	28	12
NW17	Idared	0	4		
NW17	Jonagold	32	0	24	4
NW17	Melrose	24	0		
NW17	Pinova	8	0		
***Südpfalz***					
47e	Golden Delicious			52	0
KKA1	Gala	40	0	4	0
KKA1	Jonagold	28	0		
IlbA1	unknown	20	8		
IlbA3	unknown			4	0
IlbA7	unknown			8	0
LeiA4	Delbarestivale	4	0		
LeiA4	Gala	16	0		
LeiA4	Pilot	0	4		
LeiA4	Pinova	4	0		
LeiA7	Braeburn	12	0	8	4
LeiA7	Celest	16	4		
LeiA7	Gala Royal	24	0	28	0
LeiA7	Pilot	20	0	4	0
LeiA7	Rubinette	16	0		
**total**		**672**	**72**	**284**	**132**

**Table 2:** Overview and comparison of the parameters of the used hyperspectral imaging sensors. VNIR 1800 and SWIR 384 are used in a dual-camera setup for AP-ITP-1; VNIR 1600 and SWIR 320m-e are used for AP-ITP-2, AP-ITP-3 and AP-FTS.

	VNIR 1800	VNIR 1600	SWIR 384	SWIR 320m-e
Spectral range [nm]	400–1000	416–992	930–2500	968–2497
Spatial pixels	1800	1600	384	320
Spectral bands	186	160	288	256
Spectral sampling [nm]	3.26	3.6	5.45	6
Framerate [fps]	260	135	400	100
Radiometric quantization [bit]	16	12	16	14

**Table 3:** Selected spectral indices for assessment of tree health. The abbreviation R800 denotes reflectance value in band with central wavelength of 800 nm.

Spectral Index	Definition	Reference
Normalized difference vegetation index (NDVI)	(R800 − R670)/(R800 + R670)	Huang et al. (2020) [39]
Normalized difference red edge (NDRE) ^1^	(R790 − R720)/(R790 + R720)	Davidson et al. (2022) [40]
Physiological reflectance index (PRI)	(R531 − R570)/(R531 + R570)	Kohzuma et al. (2018) [41]
(Canopy) chlorophyll content index (CCI)	NDRE/NDVI	El-Shikha et al. (2008) [42]
Green leaf index (GLI)	(2 × R550 − R650 − R440)/(2 × R550 + R650 + R440)	Gobron et al. (2000) [43]

^1^ provided for definition of CCI.

**Table 4:** Comparison of selected regression algorithms for prediction of qPCR values from spectral imaging data (experiment AP-ITP-1). RReliefF(20) using PCA, 10 features and Gaussian Process Regression performs best (bold font).

Method	Hyperparameters	Preselection	PCA	Features	RMSE
Linear regression		RReliefF (20)	x	10	17.76
		none	x	10	16.867
Ensemble of regression trees	Bagging (Random Forest)	RReliefF (20)	**x**	10	17.596
		none	x	5	19.793
Support Vector Regression	Linear kernel	RReliefF (20)	x	10	18.143
		none	**x**	10	16.867
	Gaussian kernel (scale 3.2)	RReliefF (20)	x	10	16.345
		MRMR (20)	x	10	16.698
Gaussian Process Regression	Exponential GPR	**RReliefF (20)**	**x**	**10**	**14.491**
		none		186	17.968
		none	x	5	17.438
		none	x	10	15.938
		F-test (20)	x	10	18.5
		MRMR (20)	x	10	16.195
		RReliefF (20)		20	18.138

**Table 5:** Confusion matrix (10-fold cross-validation) of best-performing classification method (rRBF with relative Euclidian distance) for pixelwise classification (accuracy 0.971) for the VNIR and SWIR data of the AP-ITP experiments.

		Infected	Healthy
**VNIR**	Infected	0.983	0.017
	Healthy	0.041	0.959
		Infected	Healthy
**SWIR**	Infected	0.972	0.028
	Healthy	0.029	0.971

**Table 6:** Overview of the classification performance for the 2-class problem (healthy vs. AP-infected) in stratified dataset AP-FTS (10-fold cross-validation, mean accuracy). Accuracy of best performing algorithms shown in bold face.

Method	Hyperparameters	VNIR 2019	VNIR 2020	SWIR 2019	SWIR 2020
Decision Tree	GINI diversity index, max 100 splits	0.639	0.66	0.624	0.688
	GINI diversity index, max 20 splits	0.645	0.656	0.617	0.676
	GINI diversity index, max 4 splits	0.603	0.641	0.597	0.665
Ensembles of decision trees	Bagging (Random Forest)	0.673	**0.7**	0.645	**0.731**
	Boosting	0.665	0.676	0.642	0.697
Support Vector Machines	linear kernel	0.654	0.662	0.634	0.71
	quadratic kernel	0.664	**0.7**	**0.685**	0.728
	cubic kernel	0.653	0.676	0.646	0.713
	Gaussian kernel	**0.678**	**0.7**	0.65	0.708
Neural Networks 10 neurons per layer, ReLU, Bayesian optimization	1 layer	0.631	0.678	0.662	0.721
	MLP 2 layers	0.652	0.676	0.66	0.728
	MLP 3 layers	0.644	0.682	0.659	0.723

**Table 7:** Overview of the classification results for the 2-class problem (healthy vs. AP-infected) in stratified dataset AP-FTS. Best performance is marked with bold font face.

Method	Hyperparameters	VNIR 2019	VNIR 2020	SWIR 2019	SWIR 2020
Decision Tree	GINI diversity index, max 100 splits	0.7	0.69	0.693	0.709
	GINI diversity index, max 20 splits	0.689	0.689	0.676	0.723
	GINI diversity index, max 4 splits	0.672	0.687	0.662	0.714
Ensembles of decision trees	Bagging (Random Forest)	**0.741**	0.693	**0.751**	0.731
	Boosting	0.696	0.694	0.686	**0.734**
Support Vector Machines	linear kernel	0.654	0.689	0.67	0.733
	quadratic kernel	0.673	0.686	0.702	0.729
	cubic kernel	0.599	0.537	0.7	0.715
	Gaussian kernel	0.738	**0.702**	**0.751**	0.733
Neural Networks 10 neurons per layer, ReLU, Bayesian optimization	1 layer	0.704	0.695	0.697	0.713
	MLP 2 layers	0.714	0.698	0.702	0.71
	MLP 3 layers	0.718	0.698	0.7	0.711
Improvement over spectral analysis		**+0.063**	+0.002	**+0.066**	+0.003

## Data Availability

Datasets used within this study are available upon request.
